# Synthesis of Some New 1,3,4-Oxadiazole Derivatives and Evaluation of Their Anticancer Activity

**DOI:** 10.1021/acsomega.3c07776

**Published:** 2023-12-13

**Authors:** Leyla Yurttaş, Asaf Evrim Evren, Aslıhan Kubilay, Mehmet Onur Aksoy, Halide Edip Temel, Gülşen Akalın Çiftçi

**Affiliations:** † Faculty of Pharmacy, Department of Pharmaceutical Chemistry, Anadolu University, 26470 Eskişehir, Turkey; ‡ Department of Pharmacy Services, Vocational School of Health Services, Bilecik Şeyh Edebali University, 11000 Bilecik, Turkey; § Faculty of Pharmacy, Department of Biochemistry, Anadolu University, 26470 Eskişehir, Turkey

## Abstract

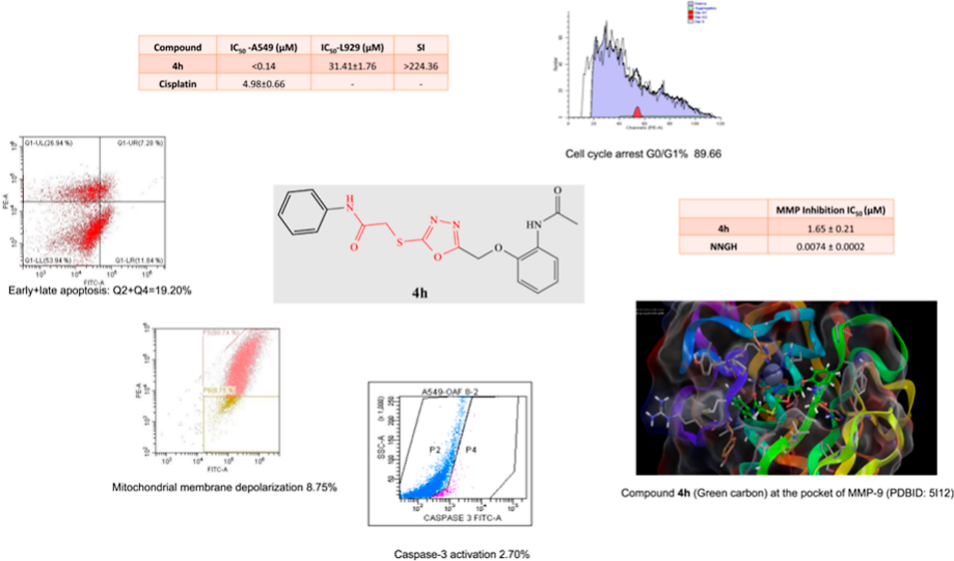

In this work, some new 2-[(5-((2-acetamidophenoxy)methyl)-1,3,4-oxadiazol-2-yl)thio]acetamide derivatives (**4a-4l**) were synthesized and studied for their anticancer activity. Twelve new compounds were tested on the A549 human lung cancer cell line, C6 rat glioma cell line, and L929 murine fibroblast cell line. Compounds **4f, 4i, 4k**, and **4l** (IC_50_: 1.59–7.48 μM), and especially **4h** (IC_50_: <0.14 μM), exhibited excellent cytotoxic profile on A549 with selectivity. Compounds **4g** and **4h** showed remarkable antiproliferative activity on the C6 cell line with IC_50_ values of 8.16 and 13.04 μM, respectively. The compounds with the lowest IC_50_ value on the A549 cell line (**4f, 4h, 4i, 4k**, and **4l**) were further studied to determine the mechanism of action. These compounds were found to induce apoptosis with a higher ratio (16.10–21.54%) than that of the standard drug cisplatin (10.07%). Compound **4f** displayed mitochondrial membrane depolarization and caspase-3 activation at most, whereas compounds **4h** (89.66%) and **4i** (78.78%) had outstanding retention rates in the G0/G1phase of the cell cycle (cisplatin 74.75%). Compounds **4f, 4g, 4h**, and **4l** exhibited matrix metalloproteinase-9 (MMP-9) inhibition higher than 75% at 100 μg/mL; even IC_50_ values were found to be 1.65 and 2.55 μM for **4h** and **4l**. In addition, *in silico* physicochemical properties of the compounds and molecular docking interaction of compound **4h** on the MMP-9 enzyme were evaluated; the desired and expected results were obtained.

## 1 Introduction

Cancer is a progressive and aggressive disease that can affect many organs and tissues. In this disease, which causes the death of millions of people every year, limitations in treatment arise due to reasons such as late diagnosis, type and location of tumor tissue, and resistance to existing drugs. Developed anticancer drugs target enzymes, genes, or signals that function in key steps such as angiogenesis, apoptosis, tumor invasion, and metastasis.^1,2^ MMP-9, matrix metalloproteinase-9, is an enzyme responsible for supporting the cell and tissue structure. Several studies have indicated that MMPIs (matrix metalloproteinase inhibitors) have the potential to impede cell proliferation by promoting apoptosis.^3^

Heterocyclic compounds constitute 70% of the anticancer medications approved by the FDA between 2010 and 2020.^4^ Heterocyclic rings exhibit their biological activity in different pathways owing to their ability to engage in various intermolecular interactions such as hydrogen bond donor/acceptor characteristics, metal coordination complexes, Π-stacking interactions, and van der Waals and hydrophobic forces.^5,6^ Among the heterocyclic rings, 1,3,4-oxadiazole derivatives with an azole structure are significant heteroaromatic structures in terms of their broad biological activity profiles and their presence in the structure of many drugs.^7−11^ Numerous compounds have been synthesized, especially related to the cytotoxic activity potential of this ring, and it has been determined that it has high anticancer activity.^12−15^ According to the reported literature, 1,3,4-oxadiazole derivatives exhibit their anticancer activities by blocking various enzymes and growth factors such as telomerase, topoisomerase, histone deacetylase (HDAC), methionine aminopeptidase, thymidylate synthase, poly(ADP-ribose) polymerase, focal adhesion kinase, thymidine phosphorylase, glycogen synthase kinase-3, caspase-3, MMP-9^16−19^ enzymes, and epidermal growth factor, vascular endothelial growth factor, and nuclear factor κB, which have specific roles in apoptosis, mitogenesis, angiogenesis, and metastasis pathways in tumors.^20^ The 1,3,4-oxadiazole ring and amide, ester, and carbamate functional groups are bioisosteres and cause an increase in pharmacological activity thanks to the hydrogen bonds they form with the receptor in the chemical structure to which they are attached. The instability of amides is regulated through metabolic degradation due to the pharmacokinetic properties of the oxadiazole ring in aqueous environments.^21−24^ In the studies of Valente et al., it was observed that cytotoxic and apoptotic activity increased at a comparable rate as a result of bioisosteric displacement of the 1,3,4-oxadiazole ring and amidic pharmacophores such as hydroxamate and 2-aminoanilide, which are known to be effective in the inhibition of HDAC in anticancer therapy.^25,26^ Oxadiazoles also provide an increase in lipophilicity and, ultimately, facilitate the transmembrane diffusion of the drug.^27^ Furthermore, the oxadiazole ring serves as a crucial component of the pharmacophore by binding to the ligand. In certain instances, it functions as a straight aromatic linker to guarantee that the molecule is oriented correctly and modulates the molecular properties by placing them around the molecule.^28,29^

**Figure 1:**
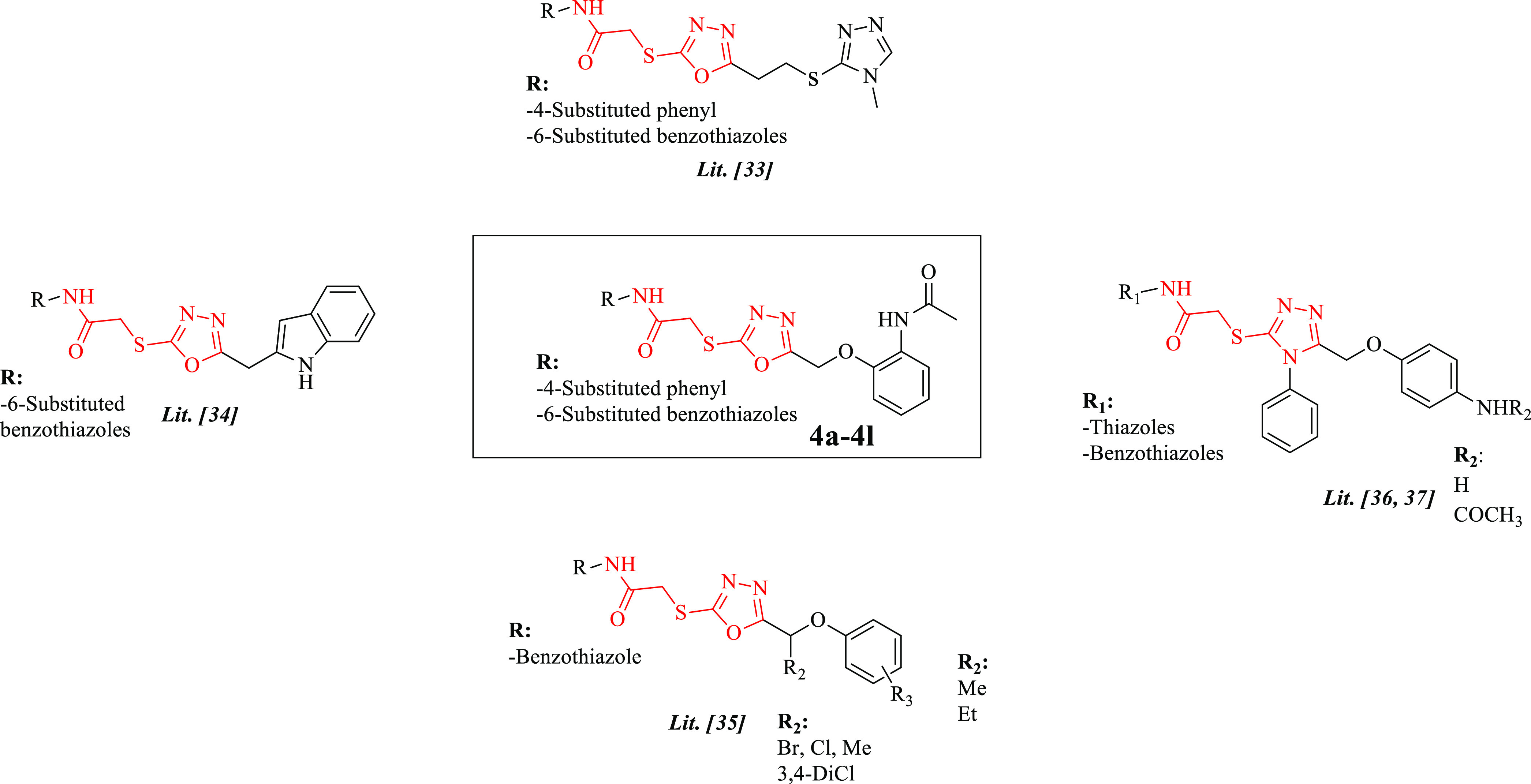
*N*-(Benzothiazol/thiazol-2-yl) or *N-*(substituted phenyl) including the 2-[(5-substituted-1,3,4-oxadiazol-2-yl)thio]acetamide framework in some studies and **4a-4l** compounds.

In addition, antiproliferative activity has also been observed in heteroaryl acetamides.^30−32^ Specifically, our research group^33^ and other researchers^34,35^ have frequently reported the presence of the *N*-(benzothiazol-2-yl)-2-[(5-substituted-1,3,4-oxadiazol-2-yl)thio]acetamide framework with significant apoptotic activity. Furthermore, our previous studies have examined the anticancer properties of 1,2,4-triazole derivatives, which serve as nitrogenous bioisosteric analogues of the 1,3,4-oxadiazole ring. These derivatives, containing mercapto-amidothiazole/benzothiazoles, exhibited notable inhibition levels of the MMP-9 enzyme and demonstrated high antiproliferative activity.^36,37^ (Figure 1).

Based on the reported literature above and our previous studies, novel 1,3,4-oxadiazole derivatives bearing mercapto acetylamido phenyl/benzothiazoles were designed and investigated for their potential anticancer activity profile, investigating cytotoxicity, apoptosis, caspase-3, MMP-9 inhibition, cell cycle analysis, and mitochondrial membrane depolarization.

## 2 Results and Discussion

### 2.1 Chemistry

**Scheme 1:**
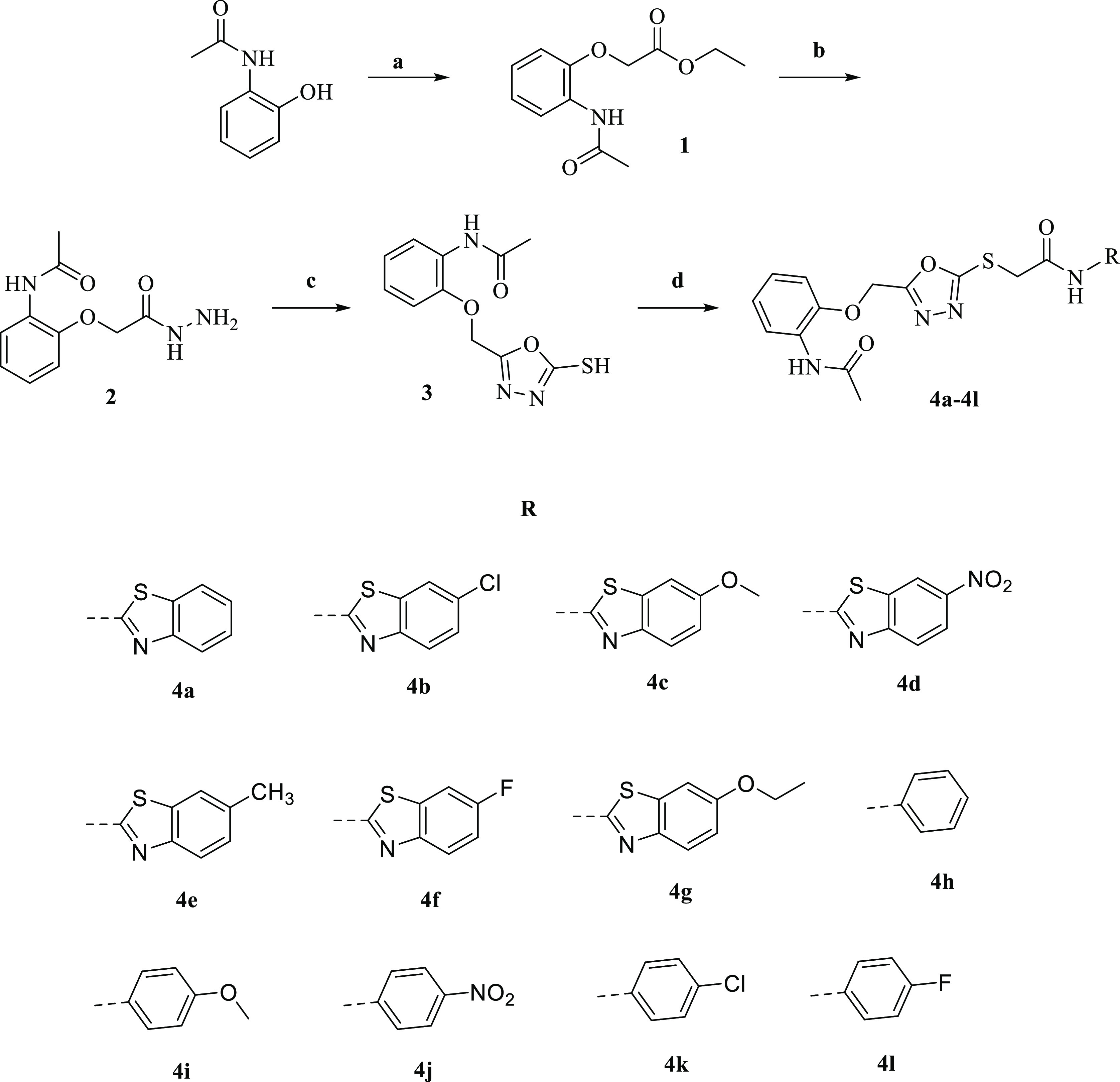
Synthesis Route of the Compounds (**4a**–**4l**) and Substituents of Derivatives Reaction conditions: (a) Ethyl 2-chloroacetate, K_2_CO_3_, acetone, reflux, 8 h. (b) N_2_H_4_·H_2_O, EtOH, r.t., 5 h. (c) CS_2_, NaOH, EtOH, 0–5 °C, then reflux, 6 h. (d) Aryl acetamides, K_2_CO_3_, acetone, r.t., 3–6 h.

This study aimed to synthesize novel 1,3,4 oxadiazole derivatives including an aryl/heteroaryl acetamido mercapto structure. First of all, ethyl 2-chloroacetate, *N*-(2-hydroxyphenyl)acetamide, and potassium carbonate were mixed in acetone, and the mixture was stirred under reflux conditions. The reaction was checked by the thin-layer chromatography (TLC) method. Following the completion of the reaction, the solvent was removed, and the raw product was filtered and cleaned with water. Crystallization of the crude product from ethanol was performed to get the pure product. The acquired intermediate, ethyl 2-(2-acetamidophenoxy)acetate (**1**), was treated with hydrazine monohydrate in ethanol. At the end of the reaction, checked by TLC, the hydrazide compound (**2**) was obtained by filtration. Freshly prepared sodium ethoxide was added to *N*-[2-(2-hydrazinyl-2-oxoethoxy) phenyl] acetamide (**2**) and mixed. Then, carbon disulfide was added to the ice bath. The mixture was boiled under reflux conditions for 6 h. The end of the reaction was confirmed by TLC. After cooling, the solution was acidified by using a solution of hydrochloric acid. After being filtered off the test medium, the precipitated portion was cleaned with water, dried, and crystallized from ethanol. At last, the resulting oxadiazole molecule (**4**) was treated with appropriate aryl acetamide derivatives to acquire the final 12 molecules (**4a**–**4l**) (Scheme 1). The targeted compounds (**4a–4l**) were obtained in pure form, and spectroscopic techniques were used to clarify their structures.

^1^H NMR, ^13^C NMR, and HRMS spectra and elemental analysis were used to elucidate the structure of the 12 synthesized materials. The σ values of detected peaks of the acetamide-NH, –NH, O–CH_2_, S–CH_2_, and –CH_3_ protons in all materials were between 9.19 and 9.43, 10.29 and 13.22, 4.67 and 5.41, 4.17 and 4.47, and 2.06 and 2.11 ppm, respectively. The signal belonging to the O–CH_3_ proton for **4c** was observed at 3.81 ppm. The peak obtained at 2.41 ppm for **4e** was a distinctive peak of the benzothiazole-CH_3_ proton. For compound **4g**, signals for the O–CH_2_–CH_3_ and the O–CH_2_–CH_3_ groups were seen at 1.35 and 4.07 ppm, respectively. Methyl proton of compound **4i** bonded to the carbonyl was detected at 3.72 ppm. Besides, multiplet peaks were detected in the aromatic region varying according to the substituents they contain in the range of 6.87–8.26 ppm. In ^13^C NMR spectra of the compounds, signals belonging to –CH_3_, S–CH_2_, and O–CH_2_ were detected at 21.47–24.39 ppm, 29.46–37.29 ppm, and 60.84–67.45 ppm, respectively. The signal seen at 56.10 ppm for compound **4c** was assigned to the O–CH_3_ carbon. For compound **4e**, the benzothiazole-CH_3_ signal was observed at 21.47 ppm. Also, signals at 15.16 and 60.87 ppm seen for compound **4g** were assigned to O–CH_2_–CH_3_ and O–CH_2_–CH_3_, respectively. The signal of carbonyl carbon was resonated at 164.31–171.85 ppm similar to all final compounds. In mass spectroscopy, peaks were identified by the compounds’ molecular weights when the HRMS of the compounds was conducted in negative ion mode. The molecular formula, which was determined in the range of ±0.4% with molecular weights, was also verified by the findings of the elemental analysis.

### 2.2 Anticancer Activity Evaluation

#### 2.2.1 Cytotoxicity

The cytotoxic activity of 12 final compounds (**4a**–**4l**) on adenocarcinoma human alveolar basal epithelial cell (A549), mouse glioma cell line (C6) and connective mouse tissue cell (L929) lines was determined and 50% inhibition concentrations (IC_50_) are presented in Table 1 in terms of μM.

**Table 1:** IC_50_ Values of the Tested Compounds against A549, C6, and L929 Cell Lines (μM) (Mean ± S.D)^a^

compounds	A549	C6	L929	SI (L919/A549)
**4a**	22.33 ± 1.67	18.53 ± 5.58	586.07 ± 22.86	26.250
**4b**	24.49 ± 5.76	19.86 ± 6.33	<7.96	<0.325
**4c**	>1000	22.12 ± 23.71	8.52 ± 0.31	<0.008
**4d**	>1000	28.34 ± 12.10	<7.80	<0.008
**4e**	43.01 ± 2.79	458.42 ± 28.21	29.85 ± 3.01	0.69
**4f**	6.62 ± 0.32	13.04 ± 3.72	19.45 ± 0.89	2.94
**4g**	<7.82	8.16 ± 0.24	<7.82	<1
**4h**	<0.14	>1000	31.41 ± 1.76	>224.36
**4i**	1.59 ± 0.44	Nd	14.63 ± 1.59	9.20
**4j**	<8.80	>1000	<8.80	<1
**4k**	7.48 ± 0.58	>1000	38.19 ± 4.91	5.11
**4l**	1.80 ± 0.12	>1000	396.63 ± 10.19	220.35
**cisplatin**	4.98 ± 0.66	Nt	Nt	

a The IC_50_ values were reported as the average of three independent determinations and their unit is μM. Nt: not tested; Nd: not determined. SI: Selectivity index calculated by the following formula (SI = IC_50_ on normal cells/IC_50_ on cancer cells).

Cisplatin was used as a standard drug, and IC_50_ was found to be 4.98 μM against the A549 cell line. Except compounds **4c** and **4d** (>1030.93 μM), all compounds exhibited high antiproliferative activity ranging from 1.59 to 43.01 μM against the A549 cell line. Compounds **4i** and **4l** showed two times higher cytotoxic activity than cisplatin with IC_50_ values of 1.59 and 1.80 μM, respectively. The IC_50_ dose of compound **4h**, namely, 2-[(5-((2-acetamidophenoxy)methyl)-1,3,4-oxadiazol-2-yl)thio]-*N*-phenylacetamide was even lower than 0.14 μM. When the cytotoxicity of L929 in the healthy cell line was examined to determine the selectivities of these three compounds, it was seen that the **4h, 4i**, and **4l** compounds stand out as bright molecules with a nontoxic profile and a high selectivity index (SI), especially **4h**. In addition, compounds **4f** (IC_50_: 6.62 μM) and **4k** (IC_50_: 7.48 μM) show high potential, followed by compound **4a** (IC_50_: 22.33 μM), selectively. Among them, the SI of compounds **4a, 4h, 4i, 4k**, and **4l** was found to be high, especially the SI of compounds **4h** and **4l** was calculated to be more than 200. Compounds **4c** and **4d** failed to kill half of the A549 cells at the highest concentration tested; however, compounds **4g** and **4j** exhibited greater than 50% inhibition at the lowest concentration tested. Since these compounds are toxic to L929, IC_50_ doses were not calculated.

Evaluation of the values of the compounds against the C6 cell line shows that compound **4g** has an IC_50_ of 8.16 μM and **4f** has an IC_50_ of 13.04 μM. The selectivity of the compounds is weak. When the other compounds were examined, it was identified that the compounds **4a**–**4d** exhibited cytotoxicity between 18.53 and 28.34 M, and only compounds **4a** and **4f** among these compounds showed a nontoxic profile.

The compounds are structurally divided into two groups: those containing substituted benzothiazole (**4a**–**4g**) and those containing substituted phenyl (**4h**–**4l**). Compound **4f** containing the 6-fluoro substituent is the most active compound on A549 among the benzothiazoles, and its SI is around 3. This situation appears to be similar and better for the other compound containing the fluoro substituent, **4l**, with an IC_50_ value lower than that of **4f** and a SI of 220 higher than it. When evaluated in general, it is seen that phenyl-containing derivatives are more effective than benzothiazole-containing derivatives. The high toxicity (<7.96 μM) of **4b** (6-chlorobenzothiazole), **4d** (6-nitrobenzothiazol), **4g** (6-ethoxybenzothiazole), and **4j** (4-nitrophenyl) compounds on healthy cells may be associated with the lipophilic character^38^ of these substituents and their toxicity in particular, NO_2_ substituent.^39^

#### 2.2.2 Apoptosis Induction

**Figure 2:**
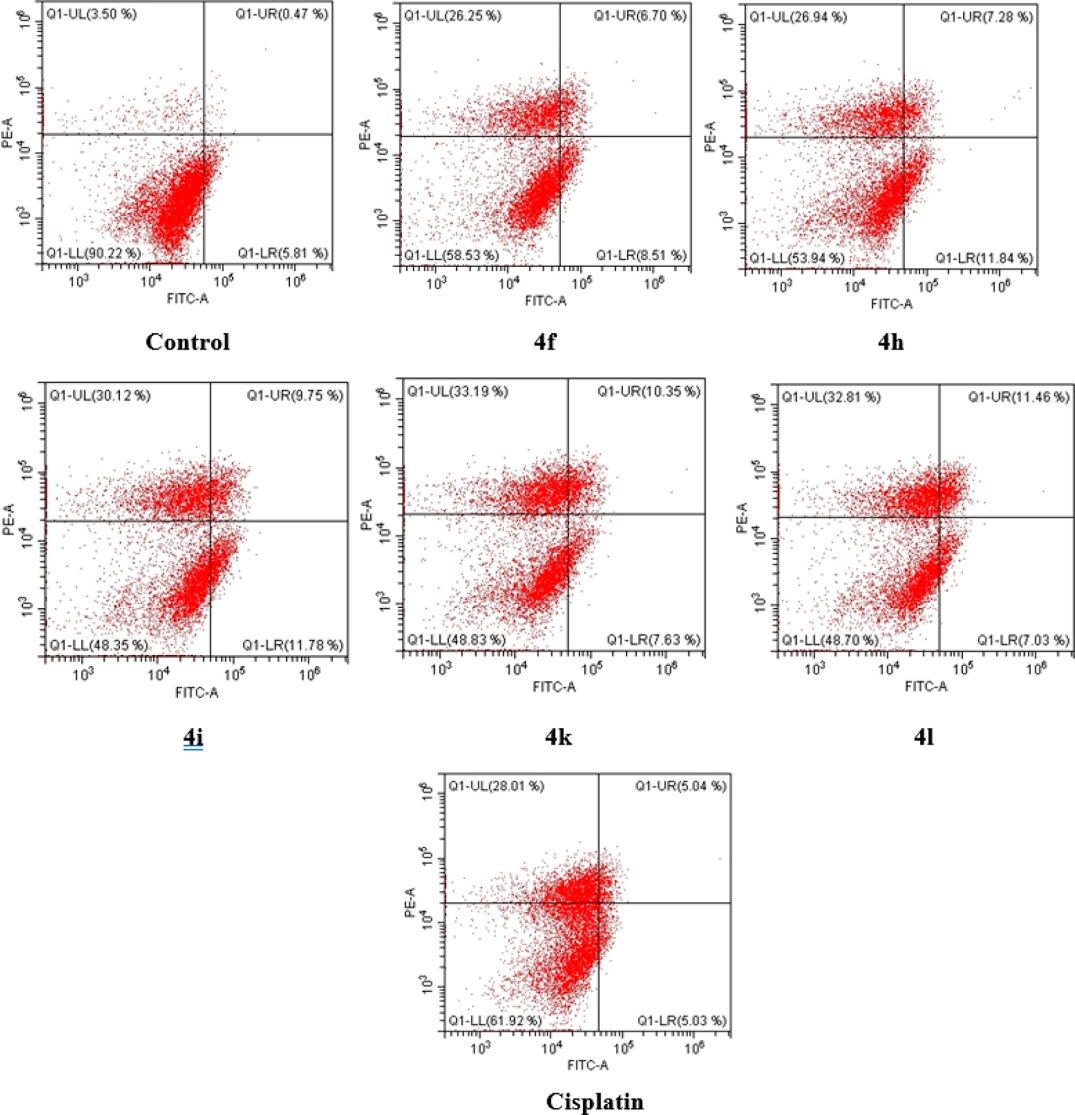
Flow cytometric analysis of A549 cells treated with IC_50_ values of compounds **4f**, **4h**, **4i**, **4k**, **4l**, and cisplatin. Each sample underwent at least 10,000 cell analyses and quadrant analysis.

During an organism’s life cycle, apoptosis is a closely controlled mechanism that confers advantages over necrosis, which is a form of catastrophic cell death caused by acute cellular injury.^40^ When the genetic basis of apoptosis is disrupted by mutation with a metabolic or developmental program, defects cause a range of human diseases, from neurodegenerative disorders to malignancy.^41^ Cell death from the apoptosis pathway is a planned and desired biochemical process, and it is targeted to prevent tumor formation, development, and metastasis. To determine the compounds with a high inhibition concentration and selective effect on the lung cancer cell line, the experiment was carried out in flow cytometry using the Annexin V kit to test how they cause the death of tumor cells. It was determined whether the most effective compounds **4f**, **4h**, **4i**, **4k**, and **4l** inhibit cells through apoptosis or necrosis, and the results are presented in Table 2 as percentages and in Figure 2 as diagrams for each compound and cisplatin. Compounds were administered at the determined IC_50_ doses, and the results were evaluated after 24 h of incubation. It has been determined that the tested compounds cause cell death through apoptosis at a high rate. While cisplatin caused apoptosis at a rate of 10.07% (early + late), the aforementioned compounds caused apoptosis-induced cell death in the range of 16.10–21.54%. Compounds **4i** (21.54%) and **4h** (19.20%) cause cell death from the apoptotic pathway with the highest percentages, while compounds **4k** (33.19%) and **4l** (32.81%) cause death from the necrotic pathway at the highest level, which are also higher than that exhibited by cisplatin. When the percentages of apoptotic cells were examined, the only derivative including benzothiazole compound **4f** induced apoptosis with the lowest percentage among the tested compounds.

**Table 2:** Apoptotic Rates of Active Compounds (**4f**, **4h**, **4i**, **4k**, **and 4l**) and Cisplatin on A549 Cell Lines^a^

compounds	Q1	Q2	Q3	Q4	Q2 + Q4
**control**	3.5	0.47	90.22	5.81	6.12
**4f**	26.25	6.7	58.53	8.51	16.10
**4h**	26.94	7.28	53.94	11.84	19.20
**4i**	30.12	9.75	48.35	11.79	21.54
**4k**	33.19	10.35	48.83	7.63	17.98
**4l**	32.81	11.46	48.70	7.03	18.49
**cisplatin**	28.01	5.04	61.92	5.03	10.07

a Q1: necrotic cells, Q2: late apoptotic cells, Q3: viable cells, Q4: early apoptotic cells, Q2 + Q4: early and late apoptotic cells.

#### 2.2.3 Inhibition of Caspase-3

**Figure 3:**
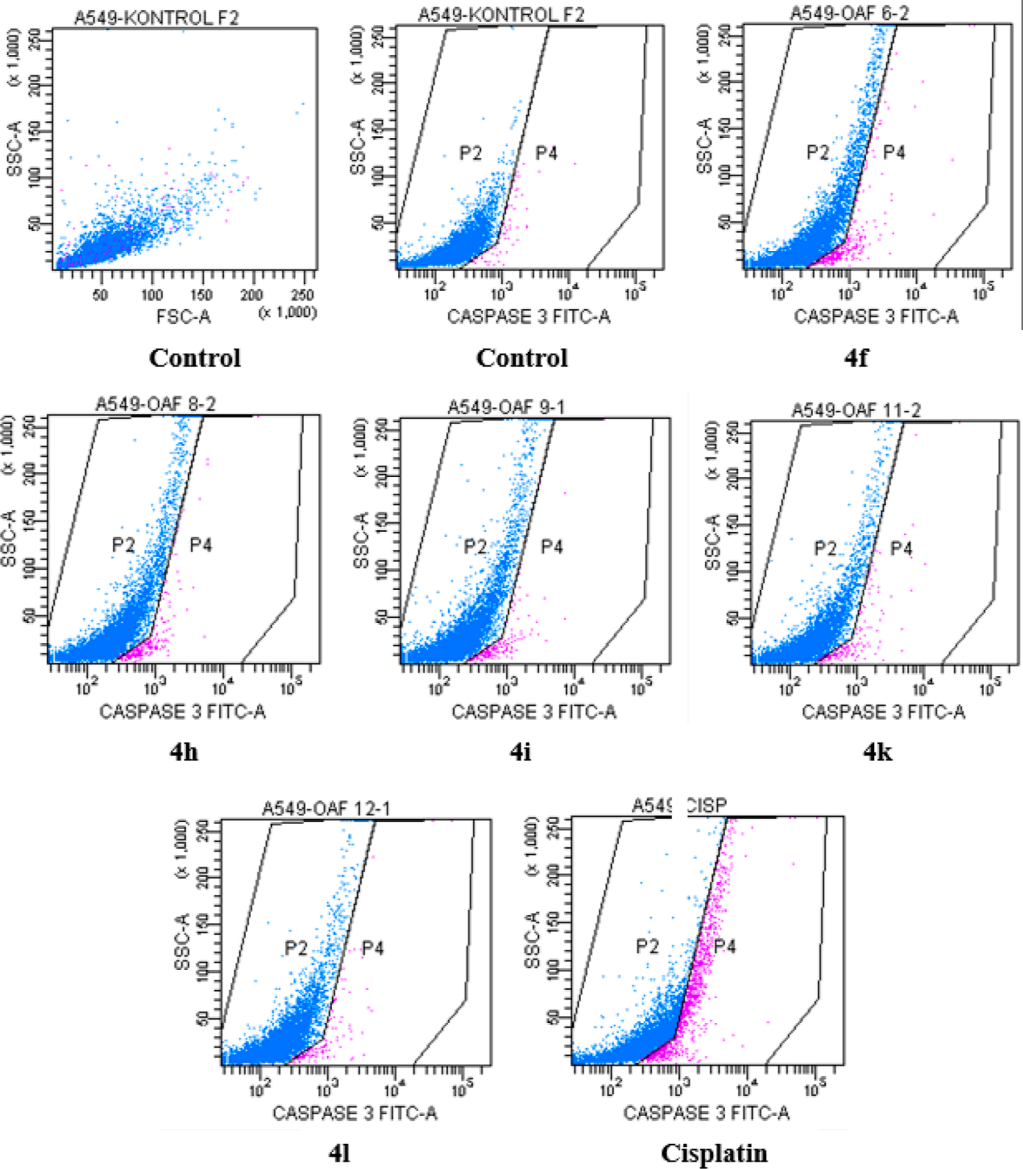
Caspase-3 activity results after 24 h incubation of A549 cells with compounds **4f**, **4h**, **4i**, **4k**, **4l**, and cisplatin.

Caspases are enzymes that play a role in programmed cell death, the absence of which, together with many factors, causes tumor development. Many anticancer agents activate caspase-3, resulting in cell death in tumors. However, the overactivation of caspase-3 causes neurodegenerative diseases and promotes carcinogenesis.^42^ Caspase-3 activation caused by compounds **4f**, **4h**, **4i**, **4k**, **4l**, and cisplatin was determined by flow cytometry, and positive and negative cells are given as percentages. The findings and diagrams are displayed in Table 3 and Figure 3, respectively. Accordingly, none of the compounds caused caspase-3 activation as much as cisplatin. While 0.8% activation of caspase-3 (positive cells) was observed in the control group, activation was observed in the range of 1.4–3.4% in all tested compounds. Of the compounds, the derivative (**4f**) containing 6-fluorobenzothiazole caused the highest activation with a value of 3.4%.

**Table 3:** Percent of Quadrant Analysis of Active Caspase-3 Phycoerythrin Staining by Flow Cytometry of A549 Cells Treated, with IC_50_ of the Compounds^a^

compounds	% (−)	% (+)
**control**	99.5	0.8
**4f**	97.1	3.4
**4h**	97.5	2.7
**4i**	98.2	1.9
**4k**	98.5	1.8
**4l**	98.8	1.4
**cisplatin**	90.0	11.3

a % (+): caspase-3 activity positive (+) cells (%) and % (−): caspase-3 activity negative (−) cells.

#### 2.2.4 Mitochondrial Membrane Depolarization

**Figure 4:**
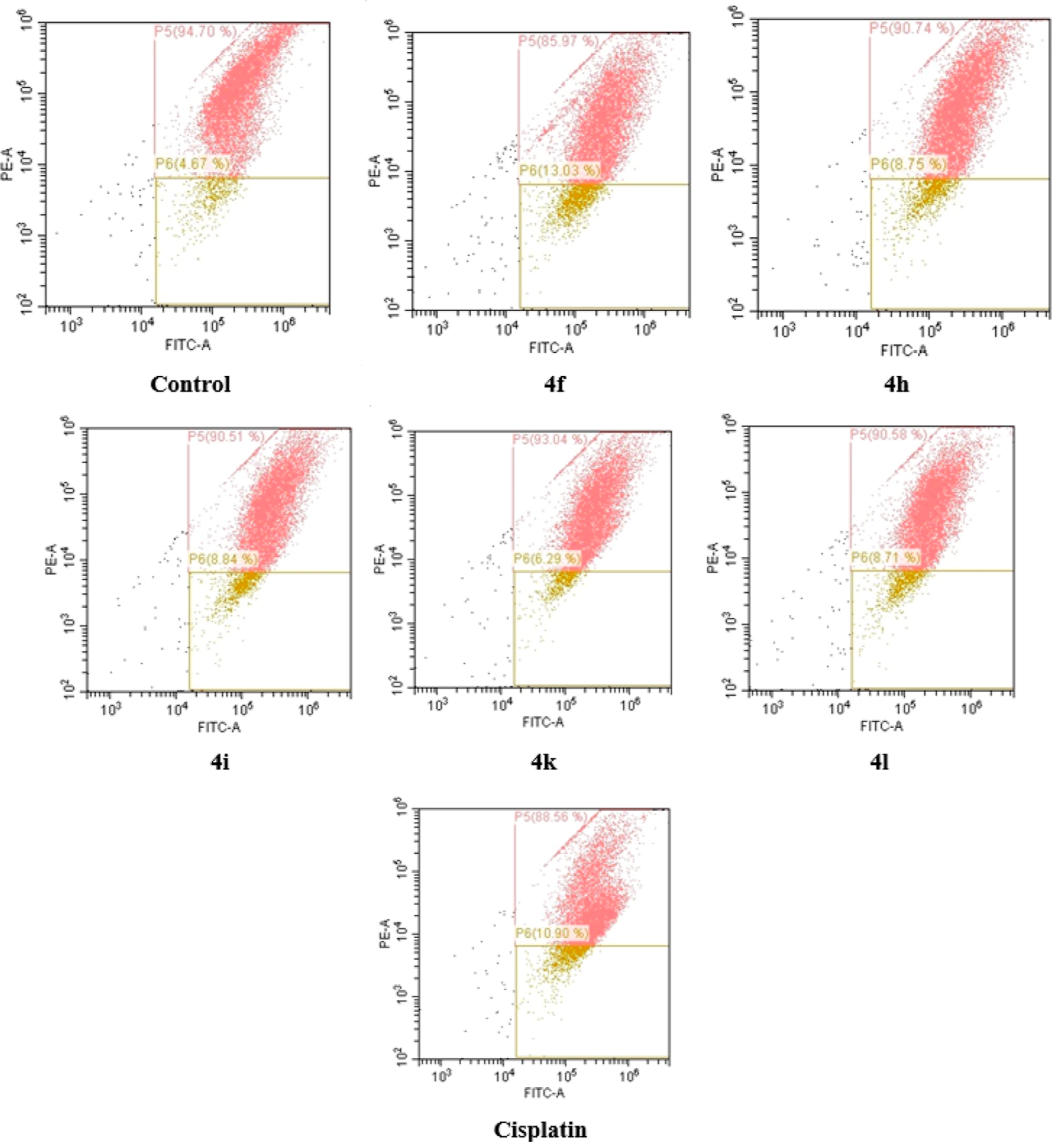
Percentages of mitochondrial membrane polarization/depolarization of A549 cells after 24 h incubation with compounds **4f**, **4h**, **4i**, **4k**, **4l**, and cisplatin.

Mitochondrial membrane potential is an important measure of the absence of mitochondrial activity. The loss of mitochondrial membrane potential may cause the release of apoptotic factors that cause cell death.^43^ Compounds **4f, 4h, 4i, 4k**, **4l**, and cisplatin were tested to identify the mitochondrial membrane potential. The test results are listed in Table 4 and Figure 4. It was observed that the compounds increased the percentage of the mitochondrial membrane depolarized cells compared to control cells. Among them, compound **4f** exhibited the highest depolarization ratio of 13.03% compared to those of other compounds. However, the depolarization effect of the tested compounds was lower than the effect of cisplatin (22.06%).

**Table 4:** Mitochondrial Membrane Potential Polarization

compounds	polarization %	depolarization %
**control**	94.70	4.67
**4f**	85.97	13.03
**4h**	90.74	8.75
**4i**	90.51	8.84
**4k**	93.04	6.29
**4l**	90.58	8.71
**cisplatin**	76.67	22.36

#### 2.2.5 Cell Cycle Analysis

**Figure 5:**
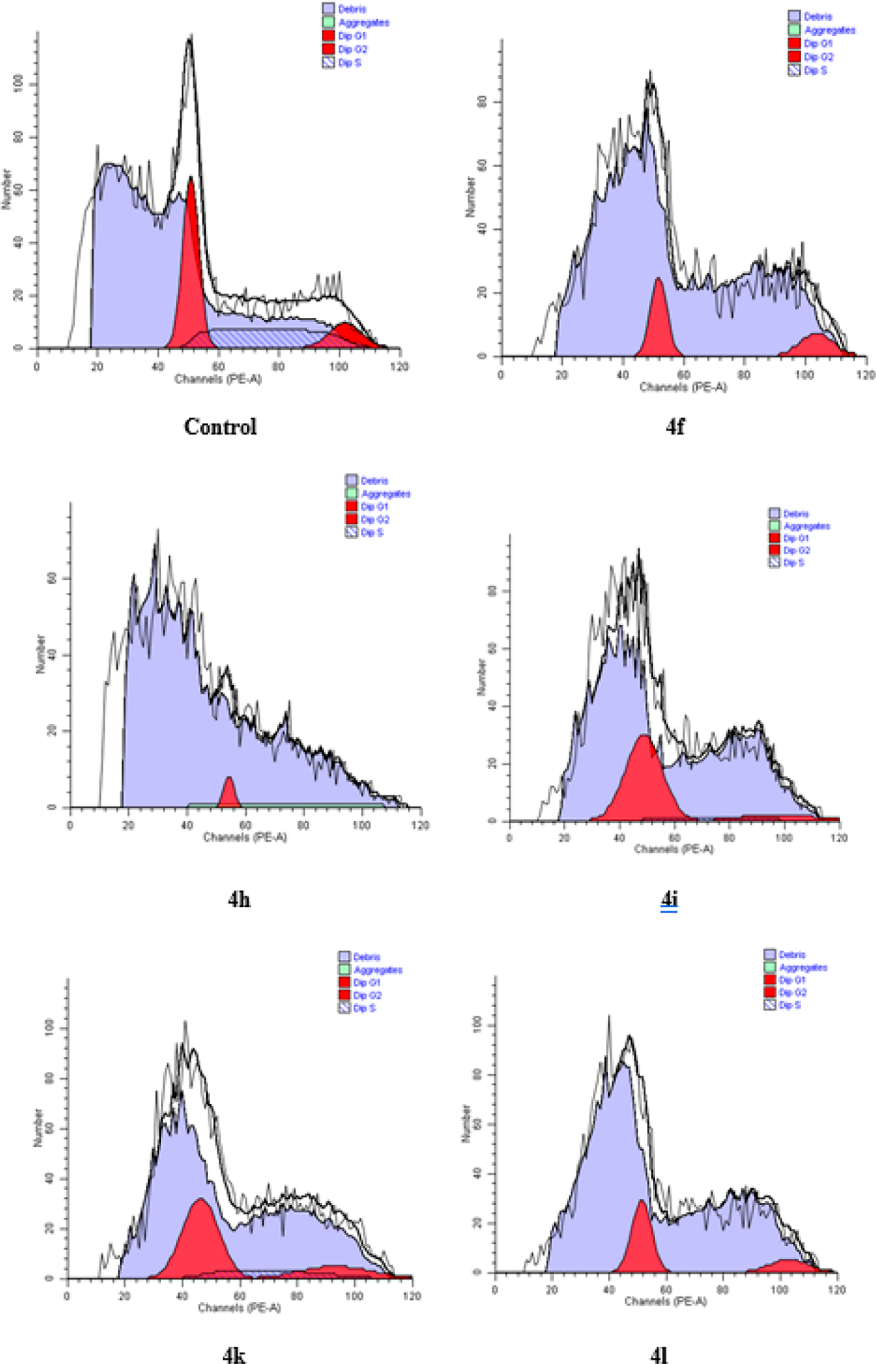
Percentages of cell cycle involvement of A549 cells after 24 h incubation with compounds **4f**, **4h**, **4i**, **4k**, **4l**, and cisplatin.

The cell goes through the G1, G2, S, and M phases in the process of replicating its genetic material and dividing it into two to reproduce. Chemotherapeutic agents can be cell cycle-specific or nonspecific. The G1 phase is the phase in which protein synthesis is the most, and transcription takes place. While few chemotherapeutic agents act at this stage, many agents act in the S (synthesis) phase, where replication takes place. The G2 phase is the phase in which RNA is produced and the M phase is the phase where mitosis and cell division occur.^44^ In this study, 24 h life cycles of A549 cells treated with five compounds (**4f, 4h, 4i, 4k**, and **4l**) were analyzed by flow cytometry, and the results are presented in Table 5 and Figure 5. Compound **4h** with 89.66% and **4i** with 78.78% caused remarkable rates of the G0/G1 phase retention, which were higher than that of cisplatin (74.75%), which is a desirable and preferred feature of anticancer agents. Compounds **4f** (63.53%), **4k** (63.31%), and **4l** (69.36%) also evoked greater G0/G1 retention compared to control cells (48.74%). Compounds **4f** and **4h** had no effect in the S phase, whereas the other three compounds **4i, 4k**, and **4l** showed a higher percentage effect than cisplatin but less than the control group. Compound **41** showed a higher retention rate than cisplatin in the G2/M phase, while it was less than the control group in compounds **4h** and **4i**.

**Table 5:** Percentages of A549 Cell Cycle Analysis

compounds	G0/G1%	% S	G2/M %
**control**	48.74	37.04	14.21
**4f**	63.53	0.0	36.47
**4h**	89.66	0.0	10.34
**4i**	78.78	8.09	12.14
**4k**	63.31	18.11	18.58
**4l**	69.36	6.62	24.03
**cisplatin**	74.75	2.18	23.07

#### 2.2.6 MMP-9 Inhibition

MMP-9 is a zinc-dependent enzyme that has a role in diabetes, cancer (leukemia, colorectal, lung, and breast cancers), inflammatory diseases, neurodegenerative diseases (Alzheimer’s and Parkinson’s diseases), and cardiovascular (hypertension, heart failure, and atherosclerosis) and lung diseases (bronchial asthma and chronic obstructive pulmonary disease—COPD). Inhibition of the enzyme, which has been demonstrated to be efficient in processes such as cancer invasion, metastasis, and angiogenesis in cancer disease, is an important treatment approach in the regression of this disease. It is also noteworthy that drugs such as marimastat, tanomastat, prinomastat, rebimastat, and neovastat, which are known as MMP-9 inhibitors for their effects on lung cancer, contain amide and/or thioether functional groups or derivatives in their structures.^45,46^ The MMP-9 enzyme inhibition was studied at a concentration of 100 μg/mL of all compounds (**4a**–**4l**), and the results are presented in percent in Table 6. Compounds **4f** (75.08%), **4g** (83.79%), **4h** (78.49%), and **4l** (75.26%) showed the highest inhibition potency on MMP-9 at concentrations of 100 μg/mL compared to other synthesized compounds. In addition to these compounds, compounds **4e** and **4j** showed more than 50% inhibition. Among the five potent compounds used in anticancer activity mechanistic studies, **4f, 4h**, and **4l** were also determined to exhibit MMP-9 inhibition, and a common result was obtained. In contrast, this was not seen in compounds **4i** and **4k.** As a standard drug, *N*-isobutyl-*N*-(4-methoxyphenylsulfonyl)glycyl hydroxamic exhibited an IC_50_ value of 0.0074 μM. The IC_50_ doses were determined to be 1.65 and 2.55 μM for compounds **4h** and **4l**.

**Table 6:** MMP-9 Inhibition Percentages of the Synthesized Compounds at 100 μg/mL and IC_50_ Concentrations^a^

	inhibition %	IC_50_		inhibition %	IC_50_
**4a**	41.40 ± 1.85		**4g**	83.79 ± 0.12	7.10 ± 1.27
**4b**	49.46 ± 1.86		**4h**	78.49 ± 3.92	1.65 ± 0.21
**4c**	30.65 ± 2.29		**4i**	40.93 ± 2.54	
**4d**	27.62 ± 2.01		**4j**	59.68 ± 2.28	22.00 ± 1.41
**4e**	68.19 ± 3.92	19.50 ± 3.18	**4k**	48.39 ± 3.56	
**4f**	75.08 ± 2.80	24.50 ± 2.12	**4l**	75.26 ± 3.73	2.55 ± 0.66
			**NNGH**	Nd	0.0074 ± 0.0002

a nd: not determined.

### 2.3 Prediction of ADME Parameters and Lipinski’s Five

To evaluate the drug-likeness availability of compounds, several physicochemical and pharmacokinetic properties such as the number of hydrogen bond acceptors (HBAs), hydrogen bond donors (HBDs), and rotatable bonds (RotB), topological polar surface area (TPSA), partition coefficient (log *P*), water solubility (log *S*), and gastrointestinal absorption (GIA) property were estimated for all compounds, as shown in Table 7. The number of HBAs was estimated between 6 and 9 and also the number of HBDs was 2. Log *P* values were predicted to be between 1.67 and 3.36. The number of RotBs was predicted to be around 10, 11, and 12. Log S was calculated and determined to be from −4.58 to −7.05, which indicates moderate and poor aqueous solubility. Compounds including benzothiazole (**4a**–**4g**) had poor solubility in water as they possessed log S smaller than −6. The other compounds containing phenyl (**4h**–**4l**) were detected as being moderately soluble in water. GIA was predicted as high for compounds **4k** and **4l**. In Lipinski’s rules,^47^ an orally active drug should have HBA <10, HBD <5, MW < 500, and log *P* < 5. There is no inconsistency in compounds **4a**–**4l** according to the Lipinski rule, except for compound **4d**. When the cytotoxic activities and estimated log S values of the compounds were analyzed, it was seen that the five most potent compounds **4f, 4hi 4i, 4k**, and **4l** were the compounds with the highest water solubility among the other compounds, except for **4f**, and there was a correlation between their biological activities. It was determined that **4h**, which was identified as the most potent compound, was the most soluble in water, with a log S value of −4.58. Of compounds **4c** and **4d**, it is noteworthy that the TPSA, which can be considered inactive, is higher than that of the other compounds. The effect is best in compounds with TPSA ≤140. When the log P values were examined, it was seen that it was between 2.38 and 3.15 for optimum activity. Compounds **4h, 4i**, and **4l** have both the lowest molecular weight and lower IC_50_ values. Although a clear correlation was not determined when the electronic character of the substituents of the compounds was evaluated, the addition of resonance and an inductive electron-withdrawing nitro group to the structure (**4j**) decreased its potential in terms of imparting toxicity in phenyl-containing derivatives (**4h**–**4l**). No consistency was seen with those containing benzothiazoles (**4a**–**4g**).

**Table 7:** Predicted Physicochemical, Pharmacokinetic, and Medicinal Chemistry Properties of Compounds **4a**–**4l**

	MW	HBA	HBD	RotB	TPSA	log *P*_o/w_	log *S*	GIA
**4a**	455	7	2	10	172.78	2.84	–6.26	low
**4b**	490	7	2	10	172.78	3.36	–6.90	low
**4c**	485	8	2	11	182.01	2.82	–6.42	low
**4d**	500	9	2	11	218.60	2.06	–7.05	low
**4e**	469	7	2	10	172.78	3.17	–6.64	low
**4f**	473	8	2	10	172.78	3.15	–6.37	low
**4g**	499	8	2	12	182.01	3.18	–6.80	low
**4h**	398	6	2	10	131.65	2.38	–4.58	low
**4i**	428	7	2	11	140.88	2.38	–4.74	low
**4j**	443	8	2	11	177.47	1.67	–5.36	low
**4k**	433	6	2	10	131.65	2.91	–5.22	high
**4l**	416	7	2	10	131.65	2.70	–4.68	high

### 2.4 Docking Study and SAR

**Figure 6:**
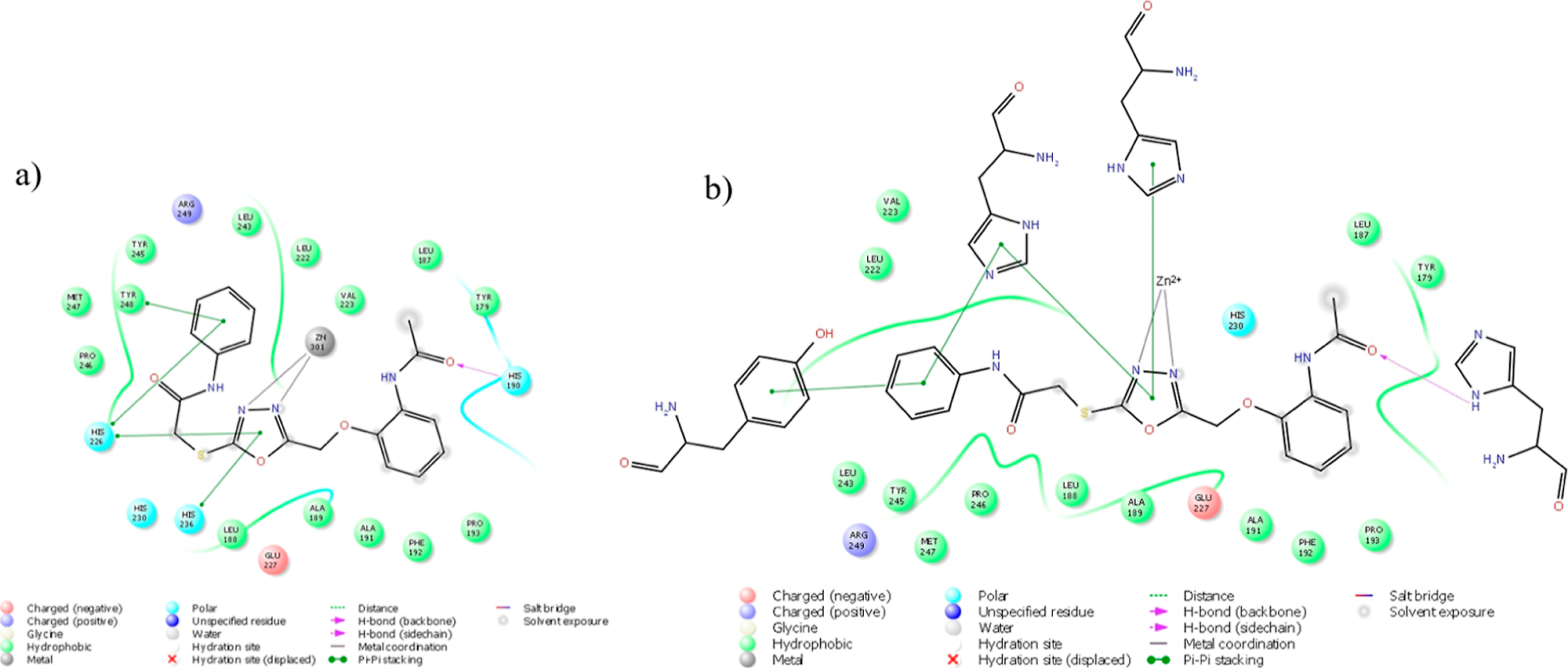
2D diagram of compound **4h** at the active pocket of MMP-9 (PDBID: 5I12). a: All residues displayed as balloon format. b: Interacted residues displayed as the open formula.

**Figure 7:**
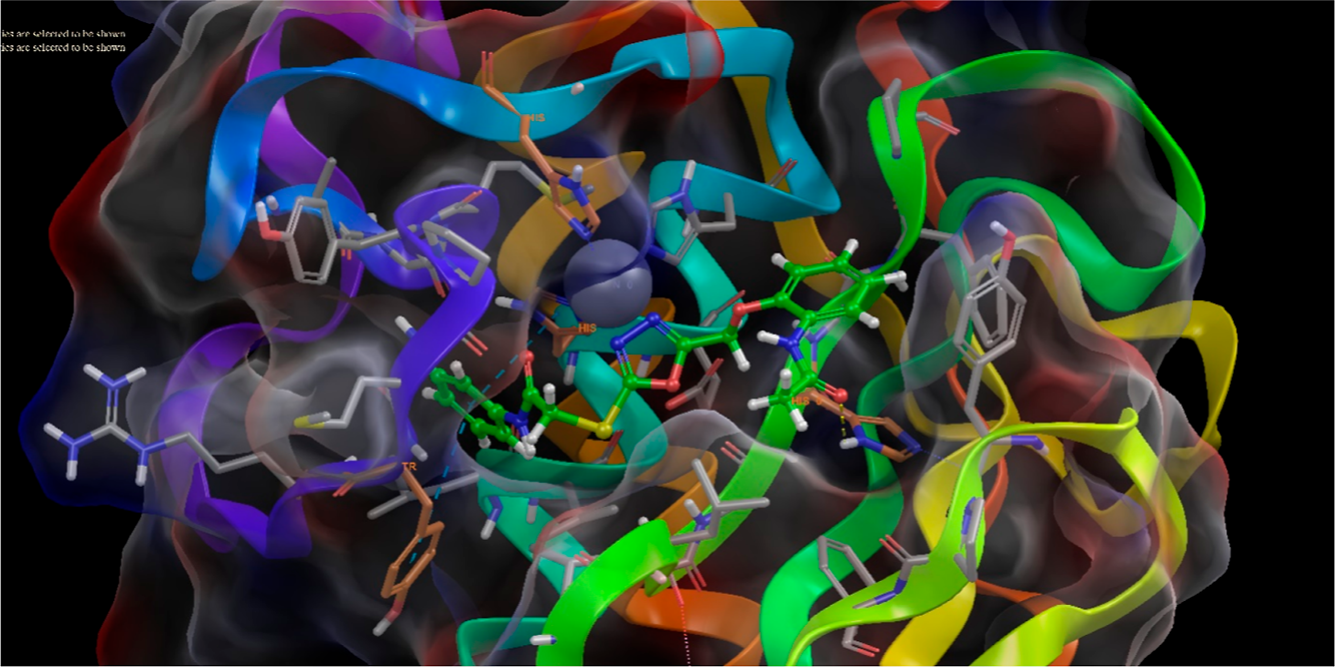
Compound **4h** (green carbon) at the pocket of MMP-9 (orange carbon for interacted residues and gray carbon for other binding side residues. PDBID: 5I12).

In light of the experimental results, first, compounds **4f** and **4h** were marked as promising anticancer agents. Unfortunately, since the SI value was calculated to be less than 3, compound **4f** seemed to be not worth searching for and utilizing as a candidate drug. Therefore, our research group marked only compound **4h** as the lead molecule to understand the structure and activity relationship. For this purpose, we performed the docking study between **4h** and the MMP-9 enzyme (PDBID: 5I12), as can be seen in Figures 6 and 7. The results showed that compound **4h** connected with His190 (H-bond), His226 (π–π stacking), His236 (π–π stacking), Tyr248 (π–π stacking) residues, and Zn301 ion (metal coordination).

**Figure 8:**
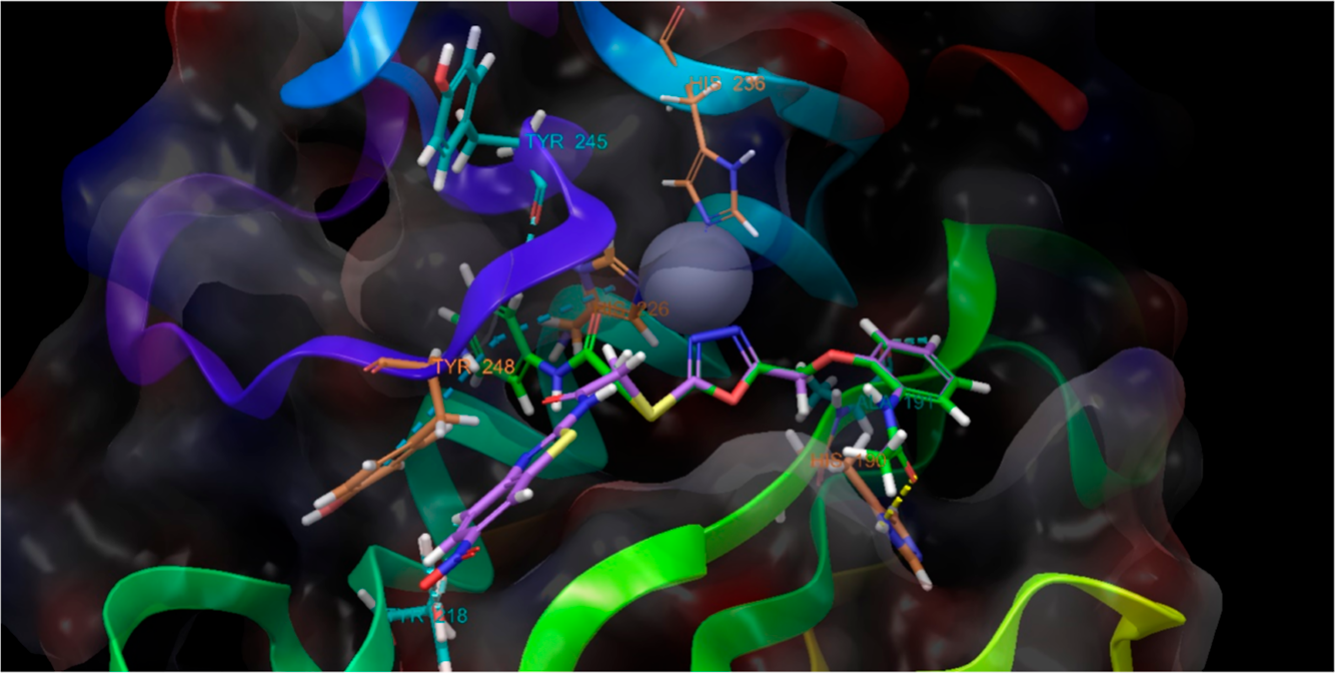
Superimposition of compounds **4d** and **4h** at the active cavity.

Not only the metal ion (Zn) but the residues (226, 230, and 236) that connect with this ion are also important.^47^ Additionally, the in vitro results of the inhibition potency on MMP-9 were observed to be similar to those of our previous studies.^35,36^ Therefore, we discussed the results together. The inhibition effect depends on ligand connections with the histamines (His226, His230, and His236, π–π interaction) and also metal ions (zinc). However, although the power of inhibition is related to the number of interactions with the same or different histamine and the number of interactions with zinc, it is obvious that it can be changed due to the ligand connections with side-chain residues via several interactions. Similar to previous docking poses,^35,36^ the benzothiazole nucleus could not fit the small pocket; hence, the rotation on the sulfur atom occurred away from this pocket and the zinc atom, and it probably resulted in the loss of connections, especially with His226 and Tyr248 residues, which was seen clearly in the superimposition of compounds **4d** and **4h** (Figure 8) at the active cavity. This is just a guess, so we hypothetically hypothesized that this could be the case considering in vitro studies.

Moreover, especially for the triazole derivatives, the triazole moiety of the ligand should be a zinc ion, because it seems vital for optimum fit between ligand and receptor. When the nitrogen atoms of **4h** (in this study) and **5f** (in ref (36)) were compared to those in **5d** (in ref (37)), it was observed that the interaction between zinc ions and third and fourth nitrogen atoms influenced the activity potency.

Generally, the combination of 2-acetamido phenoxy and oxadiazole moieties was successful in achieving an anticancer effect. Mostly, the potential differences depend on the substitutions of acetamide at the main chain. However, benzothiazole derivatives, except for compounds **4a** and **4f**, had frustrated us since some analogues did not exhibit anticancer activity (compounds **4c** and **4d**) and/or were more toxic on healthy cells (compound **4b**). Moreover, since the IC_50_ of **4a** was 10-fold that of cisplatin, only the fluorine derivative remained. Thus, it can be concluded that the existence of electron-withdrawing or -donating (except fluorine) groups decreased the anticancer activity and increased the cytotoxicity in healthy cells.

On the other hand, aniline derivatives exhibited a good SI profile and also increased the percentage of apoptotic cell death. Although their action mechanism could not be clarified by investigation of the effect on caspase-3 and mitochondrial membrane potential, the percentage of apoptotic and necrotic cells was found to be valuable. To determine the active compounds, the characteristic tests were cell cycle analysis and inhibition of MMP-9.

Briefly, aniline derivatives may be a better choice than benzothiazoles, and this may be caused by their bulky structure. 2-Amidophenoxy-oxadiazole-acetamide as a core structure, especially in this layout, has good potential and should be reviewed for future studies. Compound **4h** was determined to be the most worthwhile derivative in this study.^48^

## 3 Conclusions

We designed and synthesized 12 new oxadiazole derivatives (**4a**–**4l**) bearing a mercapto acetylamido moiety and investigated their anticancer activity. All of the compounds, except compounds **4c** and **4d**, showed better cytotoxicity against lung cancer than cisplatin and at very close rates. Compounds **4f** and **4g** showed the highest cytotoxicity against the C6 cell line. Compounds **4f, 4h, 4i, 4k**, and **4l** determined to be selective on the A549 cell line were selected and tested for further studies. All of the compounds, except for **4c** and **4d**, showed better cytotoxicity against lung cancer than cisplatin and at very close rates. Compounds **4f, 4h, 4i, 4k**, and **4l** determined to be selective were selected and tested for further studies. Compound **4h**, namely, 2-[(5-((2-acetamidophenoxy)methyl)-1,3,4-oxadiazol-2-yl)thio]-*N*-phenylacetamide showed excellent activity against A549 with IC_50_ <0.14 μM and SI <224.36. This compound induced 19.20% apoptosis, while cisplatin induced 10.07% apoptosis. The apoptosis rate was also better than that of cisplatin for the other tested compounds. It has been determined that the compounds do not cause significant caspase-3 activation and provide membrane depolarization at a lower rate than cisplatin. High retention of compounds **4h** (89.66%) and **4i** (78.78%) compared to that of cisplatin (74.75%) in the G0/G1 phase of the cell is a satisfactory result for potential anticancer drugs. When the MMP-9 inhibition of the compounds was evaluated, it was determined that the most active compounds were **4f**, **4g**, **4h**, and **4l**. The physicochemical parameters of the compounds were estimated in silico, and with one exception good results were obtained. Substituted phenyl containing derivatives (**4h**–**4l**) were found to have higher anticancer activity than benzothiazole including derivatives (**4a**–**4g**). Based on these results, molecular docking analysis of the **4h** compound was performed on MMP-9, and it was observed that compound **4h** was docked efficiently with His226 (π–π stacking), His236 (π–π stacking), and Zn301 ion (metal coordination) interactions, which are important for enzyme inhibition. In this context, compound **4h** has been evaluated as a potential and selectively effective anticancer drug candidate molecule for lung cancer, whose mechanism of action has been elucidated, partially.

## 4 Materials and Methods

### 4.1 Chemistry

The suppliers of all of the chemicals employed in the syntheses were Sigma-Aldrich Chemicals (Sigma-Aldrich Corp., St. Louis, MO, USA) and Merck Chemicals (Merck KGaA, Darmstadt, Germany). Using silica gel 60 F_254_ aluminum sheets purchased from Merck (Darmstadt, Germany), TLC was used to monitor the reactions and purities of the compounds. The uncorrected melting points of the synthesized compounds were recorded by using the MP90 digital melting point instrument (Mettler Toledo, Ohio, USA). A Bruker 300 and 75 MHz digital FT-NMR spectrometer (Bruker Bioscience, Billerica, MA, USA) was used to record the ^1^H and ^13^C NMR spectra in DMSO-*d*_6_. Splitting patterns in the NMR spectra are denoted by the following symbols: s for singlet, d for doublet, t for triplet, and m for multiplet. The reported coupling constants (*J*) were denoted as Hertz. High-resolution mass spectrometry (HRMS) studies were realized by using an LC/MS-IT-TOF system (Shimadzu, Kyoto, Japan). Elemental analyses were carried out on a Leco 932 CHNS analyzer (Leco, Michigan, USA).

#### 4.1.1 General Procedure for the Synthesis of the Ethyl 2-(2-Acetamidophenoxy)acetate (**1**)

*N*-(2-Hydroxyphenyl)acetamide (0.026 mol, 4.0 g) and ethyl 2-chloroacetate (0.029 mol, 3.57 g) were dissolved in acetone in a flask. After potassium carbonate (0.032 mol, 4.42 g) was added to the flask, the reaction mixture was refluxed for 8 h. The completion of the reaction was checked by the TLC method. After the solvent was vaporized, the raw intermediate was washed with water and filtered. Then it was recrystallized from ethanol.^49^

#### 4.1.2 General Procedure for the Synthesis of the *N*-[2-(2-Hydrazineyl-2-ox]acetamide (**2**)

Ethyl 2-(2-acetamidophenoxy)acetate **(1)** (0.022 mol, 5.21 g) and hydrazine monohydrate (0.044 mol, 2.20 g) were dissolved in 70 mL of ethanol. For 5 h, the mixture was mixed at room temperature. TLC verified that the reaction was completed. The precipitate was filtered off. Ethanol was used to recrystallize the raw material.^50^

#### 4.1.3 General Procedure for the Synthesis of the *N*-[2-((5-Mercapto-1,3,4-oxadiazol-2-yl)methoxy)phenyl]acetamide (**3**)

For 6 h, a mixture of *N*-(2-(2-hydrazinyl-2-oxoethoxy)phenyl)acetamide (**2**) (0.017 mol, 3.87 g), carbon disulfide (0.025 mol, 1.90 g), and potassium hydroxide (0.025 mol, 1.40 g) in ethanol (50 mL) was refluxed. After cooling, the mixture was acidified using a solution of hydrochloric acid. After being filtered out, the solid was cleaned with water and dried. Ethanol was used to crystallize the product.^49^

#### 4.1.4 General Procedure for the Synthesis of the 2-[(5-((2-Acetamidophenoxy)methyl)-1,3,4-oxadiazol-2-yl)thio]acetamide Derivatives (**4a**–**4l**)

*N*-[2-((5-Mercapto-1,3,4-oxadiazol-2-yl)methoxy)phenyl]acetamide (**3**) (0.014 mol, 3.73 g) and the equivalent chloro arylacetamide derivatives were dissolved in 50 mL of acetone. Potassium carbonate (0.014 mol, 1.93 g) was added into the flask, and then the mixture was stirred at room temperature for 3–6 h. The TLC method was used to verify that the reaction was completed. The substance was cleaned with water and filtered once the solvent had evaporated to a dry state. From ethanol, the main product was recrystallized.^49^

##### 4.1.4.1 2-[(5-((2-Acetamidophenoxy)methyl)-1,3,4-oxadiazol-2-yl)thio]-*N*-(benzothiazol-2-yl)acetamide (**4a**)

White or almost white color, powder. mp 189–194 °C, yield 75%. ^1^H NMR (300 MHz, DMSO-*d*_6_, ppm): δ 2.06 (s, 3H, CH_3_), 4.47 (s, 2H, S–CH_2_), 5.40 (s, 2H, O–CH_2_), 6.96 (t, *J* = 7.64 Hz, H, Ar–H), 7.05 (t, *J* = 7.70 Hz, H, Ar–H), 7.17 (d, *J* = 8.25, Hz, H, Ar–H), 7.32 (t, *J* = 7.62 Hz, H, Ar–H), 7.45 (td, *J* = 7.74 Hz, H, Ar–H), 7.77 (d, *J* = 7.95 Hz, H, Ar–H), 7.92 (d, *J* = 7.65 Hz, H, Ar–H), 7.99 (d, *J* = 7.48 Hz, H, Ar–H), 9.19 (br s, H, acetamide-NH), 12.79 (br s, H, NH). ^13^C NMR (75 MHz, DMSO-*d*_6_, ppm): δ 24.29 (CH_3_), 36.14 (S–CH_2_), 60.87 (O–CH_2_), 113.95, 121.17, 122.28 (2C), 123.17, 124.24, 124.71, 126.71, 128.58, 131.89, 148.36, 148.92, 158.15, 164.32, 164.72, 166.71(C=O), 169.00 (C=O). For C_20_H_17_N_5_O_4_S_2_ calculated: elemental analysis: % C, 52.74; % H, 3.76; % N, 15.38. Found: % C, 52.73; % H, 3.77; % N, 15.37. HRMS (*m*/*z*): [M + 1]^+^ calcd, 456.0795; found, 456.0789.

##### 4.1.4.2 2-[(5-((2-Acetamidophenoxy)methyl)-1,3,4-oxadiazol-2-yl)thio]-*N*-(6-chlorobenzothiazol-2-yl)acetamide (**4b**)

White or almost white color, powder. mp 204–206 °C, yield 71%. ^1^H NMR (300 MHz, DMSO-*d*_6_, ppm): δ 2.06 (s, 3H, CH_3_), 4.47 (s,2H, S–CH_2_), 5.40 (s, 2H, O–CH_2_), 6.95 (t, *J* = 7.77 Hz, H, Ar–H), 7.05 (t, *J* = 7.82 Hz, H, Ar–H), 7.17 (d, *J* = 8.40 Hz, H, Ar–H), 7.47 (dd, J_1_ = 2.21 Hz, J_2_ = 8.69 Hz, H, Ar–H), 7.77 (d, *J* = 8.65 Hz, H, Ar–H), 7.92 (d, *J* = 7.35 Hz, H, Ar–H), 8.14 (d, *J* = 2.14 Hz, H, Ar–H), 9.19 (br s, H, acetamide-NH), 12.88 (br s, H, NH). ^13^C NMR (75 MHz, DMSO-*d*_6_, ppm): δ 24.29 (CH_3_), 36.08 (S–CH_2_), 60.87 (O–CH_2_), 113.95 (2C), 122.01 (2C), 122.28, 123.17, 124.71, 127.07, 128.29, 128.58, 133.61, 147.84, 148.35, 158.95, 164.67, 166.91 (C=O), 168.99 (C=O). For C_20_H_16_ClN_5_O_4_S_2_ calculated: elemental analysis: % C, 49.03; % H, 3.29; % N, 14.29. Found: % C, 49.04; % H, 3.27; % N, 14.28. HRMS (*m*/*z*): [M + 1]^+^ calcd, 490.0405; found, 490.0398.

##### 4.1.4.3 2-[(5-((2-Acetamidophenoxy)methyl)-1,3,4-oxadiazol-2-yl)thio]-*N*-(6-methoxybenzothiazol-2-yl)acetamide (**4c**)

White or almost white color, powder. mp 192–196 °C, yield 69%. ^1^H NMR (300 MHz, DMSO-*d*_6_, ppm): δ 2.07 (s, 3H, CH_3_), 3.81 (s, 3H, O–CH_3_), 4.45 (s, 2H, S–CH_2_), 5.41 (s, 2H, O–CH_2_), 6.96 (t, *J* = 7.67 Hz, H, Ar–H), 7.02–7.08 (m, 2H, Ar–H), 7.17 (d, *J* = 8.26 Hz, H, Ar–H), 7.58 (d, *J* = 2.55 Hz, H, Ar–H), 7.77 (d, *J* = 8.87 Hz, H, Ar–H), 7.93 (brd, *J* = 7.51 Hz, H, Ar–H), 9.20 (br s, H, acetamide- NH), 12.66 (br s, H, NH). ^13^C NMR (75 MHz, DMSO-*d*_6_, ppm): δ 24.31 (CH_3_), 29.46 (S–CH_2_), 56.06 and 56.10 (O–CH_3_), 60.87 (O–CH_2_), 105.19 (2C), 113.95 (2C), 115.55, 121.79, 122.28, 123.16, 124.71, 128.58, 133.23, 142.99, 148.35, 156.03, 164.31, 164.74, 166.35 (C=O), 169.00 (C=O). For C_21_H_19_N_5_O_5_S_2_ calculated: elemental analysis: % C, 51.95; % H, 3.94; % N, 14.42. Found: % C, 51.94; % H, 3.95; % N, 14.43. HRMS (*m*/*z*): [M + 1]^+^ calcd, 486.0900; found, 486.0903.

##### 4.1.4.4 2-[(5-((2-Acetamidophenoxy)methyl)-1,3,4-oxadiazol-2-yl)thio]-*N*-(6-nitrobenzothiazol-2-yl)acetamide (**4d**)

Light yellow color, powder. mp 192–194 °C, yield 73%. ^1^H NMR (300 MHz, DMSO-*d*_6_, ppm): δ 2.07 (s, 3H, CH_3_), 4.46 (s, 2H, S–CH_2_), 5.41 (s, 2H, O–CH_2_), 6.95 (td, *J*_1_ = 1.20 Hz, *J*_2_ = 7.65 Hz, H, Ar–H), 7.05 (td, *J*_1_ = 1.33 Hz, *J*_2_ = 7.78 Hz, H, Ar–H), 7.17 (dd, *J*_1_ = 1.12 Hz, *J*_2_ = 8.17 Hz, H, Ar–H), 7.86 (d, *J* = 8.98 Hz, H, Ar–H), 7.91 (d, *J* = 7.66 Hz, H, Ar–H), 8.26 (q, *J*_1_ = 2.46 Hz, *J*_2_ = 8.98 Hz, Ar–H), 9.19 (br s, H, acetamide-NH), 13.22 (br s, H, NH). ^13^C NMR (75 MHz, DMSO-*d*_6_, ppm): δ 24.30 (CH_3_), 36.80 (S–CH_2_), 60.88 (O–CH_2_), 113.95 (2C), 119.34 (2C), 120.83, 122.26 (2C), 123.15, 124.71, 128.58, 132.81, 143.16, 154.32, 164.26, 165.23, 168.36 (C=O), 168.99 (C=O). For C_20_H_16_N_6_O_6_S_2_ calculated: elemental analysis: % C, 48.00; % H, 3.22; % N, 16.79. Found: % C, 48.01; % H, 3.20; % N, 16.80. HRMS (*m*/*z*): [M + 1]^+^ calcd, 501.0646; found, 501.0640.

##### 4.1.4.5 2-[(5-((2-Acetamidophenoxy)methyl)-1,3,4-oxadiazol-2-yl)thio]-*N*-(6-methylbenzothiazol-2-yl)acetamide (**4e**)

White color, powder. mp 213–216 °C, yield 71%. ^1^H NMR (300 MHz, DMSO-*d*_6_, ppm): δ 2.06 (s, 3H, CH_3_), 2.41 (s, 3H, benzothiazole-CH_3_), 4.46 (s, 2H, CH_2_), 5.40 (s, 2H, O–CH_2_), 6.96 (t, *J* = 7.66 Hz, H, Ar–H), 7.05 (td, *J* = 7.79 Hz, H, Ar–H), 7.17 (d, *J* = 7.35 Hz, H, Ar–H), 7.26 (d, *J* = 8.41 Hz, H, Ar–H), 7.66 (d, *J* = 8.22 Hz, H, Ar–H), 7.77 (s, H, Ar–H), 7.92 (d, *J* = 7.72 Hz, H, Ar–H), 9.21 (br s, H, acetamide-NH), 12.72 (br s, H, NH). ^13^C NMR (75 MHz, DMSO-*d*_6_, ppm): δ 21.47 (benzothiazole CH_3_), 24.28 (CH_3_), 36.08 (S–CH_2_), 60.84 (O–CH_2_), 113.91 (2C), 120.85, 121.85 (2C), 122.27, 123.17, 124.71, 128.05, 132.05, 133.74, 146.93, 148. 34, 157.17, 164.73, 166.48 (C=O), 169.00 (C=O). For C_21_H_19_N_5_O_4_S_2_ calculated: elemental analysis: % C, 53.72; % H, 4.08; % N, 14.92. Found: % C, 53.70; % H, 4.09; % N, 14.91. HRMS (*m*/*z*): [M + 1]^+^ calcd, 470.0951; found, 470.0955.

##### 4.1.4.6 2-[(5-((2-Acetamidophenoxy)methyl)-1,3,4-oxadiazol-2-yl)thio]-*N*-(6-fluorobenzothiazol-2-yl)acetamide (**4f**)

Beige color, powder. mp 207–209 °C, yield 70%. ^1^H NMR (300 MHz, DMSO-*d*_6_, ppm): δ 2.07 (s, 3H, CH_3_), 4.47 (s, 2H, S–CH_2_), 5.41 (s, 2H, O–CH_2_), 6.95 (t, *J* = 7.53 Hz, H, Ar–H), 7.05 (t, *J* = 7.33 Hz, H, Ar–H), 7.17 (d, *J* = 7.26 Hz, H, Ar–H), 7.30 (td, *J*_1_ = 2.69 Hz, *J*_2_ = 9.09 Hz, H, Ar–H), 7.84 (q, *J*_1_ = 4.83 Hz, *J*_2_ = 8.74 Hz, H, Ar–H), 7.89–7.92 (m, 2H, Ar–H), 9.19 (br s, H, acetamide NH), 12.82 (br s, H, NH). ^13^C NMR (75 MHz, DMSO-*d*_6_, ppm): δ 24.29 (CH_3_), 36.08 (S–CH_2_), 60.87 (O–CH_2_), 108.55 (2C), 113.94, 114.69, 122.28 (2C), 123.16, 124.71, 128.58, 148.35, 157.61, 158.10, 160.79, 164.33, 164.69, 166.77 (C=O), 168.99 (C=O). For C_20_H_16_FN_5_O_4_S_2_ calculated: elemental analysis: % C, 50.73; % H, 3.41; % N, 14.79. Found: % C, 50.72; % H, 3.42; % N, 14.80. HRMS (*m*/*z*): [M + 1]^+^ calcd, 474.0701; found, 474.0700.

##### 4.1.4.7 2-[(5-((2-Acetamidophenoxy)methyl)-1,3,4-oxadiazol-2-yl)thio]-*N*-(6-ethoxybenzothiazol-2-yl)acetamide (**4g**)

Beige color, powder. mp 208–212 °C, yield 65%. ^1^H NMR (300 MHz, DMSO-*d*_6_, ppm): δ 1.35 (t, *J* = 6.97 Hz, 3H, OCH_2_CH_3_), 2.07 (s, 3H, CH_3_), 4.07 (q, J_1_ = 6.95 Hz, J_2_ = 13.90 Hz, 2H, OCH_2_CH_3_), 4.44 (s, 2H S–CH_2_), 5.40 (s, 2H, O–CH_2_), 6.96 (t, *J* = 7.70 Hz, H, Ar–H), 7.01–7.08 (m, 2H, Ar–H), 7.17 (d, *J* = 8.27 Hz, H, Ar–H), 7.56 (d, *J* = 2.51 Hz, H, Ar–H), 7.65 (d, *J* = 8.86 Hz, H, Ar–H), 7.92 (d, *J* = 7.48 Hz, H, Ar–H), 9.19 (br s, H, acetamide-NH), 12.64 (br s, H, NH). ^13^C NMR (75 MHz, DMSO-*d*_6_, ppm): δ 15.16 (OCH_2_–CH_3_), 24.32 (CH_3_), 36.06 (S–CH_2_), 60.87 (O–CH_2_–CH_3_), 64.06 (O–CH_2_), 105.83, 113.95 (2C), 115.90 (2C), 121.80 (2C), 122.28, 123.15, 124.72, 128.57, 133.23, 142.91, 148.35, 155.96, 164.31, 164.73, 166.34 (C=O), 168.99 (C=O). For C_22_H_21_N_5_O_5_S_2_ calculated: elemental analysis: % C, 52.89; % H, 4.24; % N, 14.02. Found: % C, 52.90; % H, 4.25; % N, 14.03. HRMS (*m*/*z*): [M + 1]^+^ calcd, 500.1057; found, 500.1051.

##### 4.1.4.8 2-[(5-((2-Acetamidophenoxy)methyl)-1,3,4-oxadiazol-2-yl)thio]-*N*-phenylacetamide (**4h**)

Beige color, powder. mp 188–191 °C, yield 73%. ^1^H NMR (300 MHz, DMSO-*d*_6_, ppm): δ 2.11 (s, 3H, CH_3_), 4.17 (s, 2H, S–CH_2_), 4.67 (s, 2H, O–CH_2_), 6.92–7.09 (m, 3H, Ar–H), 7.32–7.35 (m, 2H, Ar–H), 7.44–7.54 (m, 3H, Ar–H), 7.88 (d, *J* = 7.65 Hz, H, Ar–H), 9.43 (br s, H, acetamide- NH), 10.61 (br s, H, NH). ^13^C NMR (75 MHz, DMSO-*d*_6_, ppm): δ 24.39 (CH_3_), 33.31 (S–CH_2_), 67.45 (O–CH_2_), 113.02, 121.65, 123.04, 124.87, 127.98, 128.70 (2C), 129.13 (2C), 129.55, 135.33, 148.45, 162.87, 164.26, 168.85 (C=O), 171.85 (C=O). For C_19_H_18_N_4_O_4_S calculated: elemental analysis: % C, 57.28; % H, 4.55; % N, 14.06. Found: % C, 57.29; % H, 4.56; % N, 14.07. HRMS (*m*/*z*): [M + 1]^+^ calcd, 399.1122; found, 399.1119.

##### 4.1.4.9 2-[(5-((2-Acetamidophenoxy)methyl)-1,3,4-oxadiazol-2-yl)thio]-*N*-(4-methoxyphenyl)acetamide (**4i**)

White or almost white color, powder. mp 150–152 °C, yield 67%. ^1^H NMR (300 MHz, DMSO-*d*_6_, ppm): δ 2.07 (s, 3H, CH_3_), 3.72 (s, 3H, O–CH_3_), 4.29 (s, 2H, S–CH_2_), 5.41 (s, 2H, O–CH_2_), 6.87 (d, *J* = 9.08 Hz, 2H, Ar–H), 6.97 (t, *J* = 7.69 Hz, H, Ar–H), 7.06 (t, *J* = 7.82 Hz, H, Ar–H), 7.18 (d, *J* = 8.22 Hz, H, Ar–H), 7.47 (d, *J* = 9.05 Hz, H, Ar–H), 7.92 (d, *J* = 7 Hz, H, Ar–H), 9.20 (br s, H, acetamide-NH), 10.29 (br s, H, NH). ^13^C NMR (75 MHz, DMSO-*d*_6_, ppm): δ 24.28 (CH_3_), 37.15 (O–CH_3_), 55.62 (CH_2_), 60.85 (O–CH_2_), 113.94, 114.41 (2C), 121.15 (2C), 122.27, 123.21, 124.74, 128.55, 132.21, 148.38, 155.92, 165.09 (C=O), 169.00 (C=O). For C_20_H_20_N_4_O_5_S calculated: elemental analysis: % C, 56.07; % H, 4.71; % N, 13.08. Found: % C, 56.07; % H, 4.72; % N, 13.07. HRMS (*m*/*z*): [M + 1]^+^ calcd, 429.1227; found, 429.1215.

##### 4.1.4.10 2-[(5-((2-Acetamidophenoxy)methyl)-1,3,4-oxadiazol-2-yl)thio]-*N*-(4-nitrophenyl)acetamide (**4j**)

Beige color, powder. mp 210–215 °C, yield 69%. ^1^H NMR (300 MHz, DMSO-*d*_6_, ppm): δ 2.06 (s, 3H, CH_3_), 4.39 (s, 2H, S–CH_2_), 5.41 (s, 2H, O–CH_2_), 6.96 (t, *J* = 7.63 Hz, H, Ar–H), 7.06 (t, *J* = 7.76 Hz, H, Ar–H), 7.17 (d, *J* = 8.29 Hz, H, Ar–H), 7.82 (d, *J* = 9.25 Hz, 2H, Ar–H), 7.91 (d, *J* = 8.09 Hz, H, Ar–H), 8.24 (d, *J* = 9.25 Hz, 2H, Ar–H), 9.19 (br s, H, acetamide- NH),11.03 (br s, H, NH). ^13^C NMR (75 MHz, DMSO-*d*_6_, ppm): δ 24.27 (CH_3_), 37.29 (S–CH_2_), 60.86 (O–CH_2_), 113.96, 119.38 (2C), 122.28, 123.19, 124.72, 125.57, 128.57 (2C), 142.96, 145.15, 164.22, 166.22 (C=O), 168.98 (C=O). For C_19_H_17_N_5_O_6_S calculated: elemental analysis: % C, 51.46; % H, 3.86; % N, 15.79. Found: % C, 51.44; % H, 3.87; % N, 15.78. HRMS (*m*/*z*): [M + 1]^+^ calcd, 444.0972; found, 444.0978.

##### 4.1.4.11 2-[(5-((2-Acetamidophenoxy)methyl)-1,3,4-oxadiazol-2-yl)thio]-*N*-(4-chlorophenyl)acetamide (**4k**)

Beige color, powder. mp 203–206 °C, yield 72%. ^1^H NMR (300 MHz, DMSO-*d*_6_, ppm): δ 2.07 (s, 3H, CH_3_), 4.33 (s, 2H, S–CH_2_), 5.41 (s, 2H, O–CH_2_), 6.97 (t, *J* = 7.69 Hz, H, Ar–H), 7.06 (t, *J* = 7.79 Hz, H, Ar–H), 7.18 (d, *J* = 8.12 Hz, H, Ar–H), 7.36–7.39 (m, 2H, Ar–H), 7.59–7.62 (m, 2H, Ar–H), 7.93 (d, *J* = 7.33 Hz, H, Ar–H), 9.20 (br s, H, acetamide- NH), 10.56 (br s, H, NH). ^13^C NMR (75 MHz, DMSO-*d*_6_, ppm): δ 24.28 (CH_3_), 37.20 (S–CH_2_), 60.88 (O–CH_2_), 113.97, 121.16 (2C), 122.29, 123.21, 124.74, 127.73, 128.58, 130.60 (2C), 138.04, 164.15, 165.00, 165.29 (C=O), 169.00 (C=O). For C_19_H_17_ClN_4_O_4_S calculated: elemental analysis: % C, 52.72; % H, 3.96; % N, 12.94. Found: % C, 52.73; % H, 3.97; % N, 12.95. HRMS (*m*/*z*): [M + 1]^+^ calcd, 433.0732; found, 433.0741.

##### 4.1.4.12 2-[(5-((2-Acetamidophenoxy)methyl)-1,3,4-oxadiazol-2-yl)thio]-*N*-(4-fluorophenyl)acetamide **(4l)**

Beige color, powder. mp 217–219 °C, yield 74%. ^1^H NMR (300 MHz, DMSO-*d*_6_, ppm): δ 2.07 (s, 3H, CH_3_), 4.32 (s, 2H, S–CH_2_), 5.41 (s, 2H, O–CH2), 6.94–7.06 (m, 3H, Ar–H), 7.16 (t, *J* = 8.97 Hz, H, Ar–H), 7.34–7.39 (m, 2H, Ar–H), 7.37–7.62 (m, H, Ar–H), 7.86–7.93 (m, H, Ar–H), 9.21 (br s, H, acetamide-NH), 10.58 (br s, H, NH). ^13^C NMR (75 MHz, DMSO-*d*_6_, ppm): δ 24.37 (CH_3_), 37.13 (S–CH_2_), 67.29 (O–CH_2_), 112.90 (2C), 116.07, 116.63 (2C), 121.64 (2C), 123.08, 124.90, 127.91, 130.99, 164.23, 168.85 (C=O), 171.82 (C=O). For C_19_H_17_FN_4_O_4_S calculated elemental analysis: % C, 54.80; % H, 4.12; % N, 13.45. Found: % C, 54.85; % H, 4.09; % N, 13.48. HRMS (*m*/*z*): [M + 1]^+^ calcd, 417.1027; found, 417.1027.

### 4.2 Biochemistry

#### 4.2.1 Model Cell Line

In 75 cm^2^ sterile plastic tissue culture flasks, the human lung adenocarcinoma A549 and the mouse fibroblast L929 cell lines were cultured in 90% RPMI (Sigma, Deisenhofen, Germany) media supplemented with 10% (v/v) FBS (Gibco, Paisley, UK) and adherent monolayers of penicillin/streptomycin (100 units/mL) from Gibco, Paisley, UK. These cells were cultured at 37 °C in an environment that was humidified and contained 5% CO_2_.

#### 4.2.2 Cell Viability Analysis

The MTT (3-(4,5-dimethylthiazol-2-yl)-2,5-diphenyltetrazolium bromide) method was applicated to assess the cell viability of L929, C6 and A549 cell lines against the tested compounds according to the reported data.^51^ In 96-well plates with a flat bottom, the A549, C6, and L929 cells were cultivated at a density of 5 × 103 cells per well. All the produced compounds were dissolved in DMSO, together with cisplatin as the control medication, at different concentrations ranging from 50 to 1000 μM. After addition, they were incubated for a full day in culture wells. Following the incubation period, 20 μL of phosphate-buffered saline (PBS) (Gibco, Paisley, UK) containing 5 mg/mL MTT powder (Sigma-Aldrich, St. Louis, MO, USA) was added to each well. The medium was taken out of the plate and replaced with 100 μL of DMSO in each well to dissolve the dye. This was left for 2 to 4 h under the same conditions and then left for 10 min. At the conclusion of the procedure, purple formazan, the reduction product of the MTT agent, was formed by the mitochondrial dehydrogenase enzyme of intact cells. A microtiter plate reader (BioTek Plate Reader, Winooski, VT, USA) was used to measure the cells at 540 nm. Cell viability was measured as a percentage and contrasted with that of the cells in the control group. Half-maximal inhibitory concentration 50 (IC_50_) values were determined by repeating each concentration in three wells and identifying the drug concentrations that decreased the absorbance to 50% of control values. The medium control served as the basis for calculating the percentage of viable cells.

#### 4.2.3 Determination of Early/Late Apoptosis by Flow Cytometry

The most effective antiproliferative agents in this series (**4f**, **4h**, **4i**, **4k**, and **4l**) were incubated for 24 h at IC_50_ concentrations on A549 and L929 cells. Phosphatidylserine externalization, a marker of early apoptosis, was assessed using the FITC Annexin V apoptosis detection kit (BD Pharmingen, San Jose, CA, USA) on a BD FACSAria flow cytometer. Following their collection, the A549 and L929 cells were twice washed in ice-cold PBS before being resuspended in 100 μL of binding buffer. The cells were treated with a volume of 5 μL (5 μg/mL) of Annexin V-FITC and PI, and they were incubated for 15 min at room temperature (20–25 °C) in the dark. Following that, 400 μL of binding buffer was added to the combination samples, and FACSDiva version 6.1.1 was used to analyze the samples on a BD FACSAria flow cytometer.

#### 4.2.4 Spectrofluorometric Analysis of Caspase-3 Activation

The Spectrofluorometric Caspase-3 Assay kit (BD Pharmingen, Franklin Lakes, NJ) was used to assess the activation of caspase-3. The purpose of the kit was to measure the early indicator of cells going through apoptosis, known as caspase-3 or the DEVD-cleaving activity. First, 1 × 106 cells/mL of cells were resuspended in cold cell lysis buffer, rinsed with PBS, and then allowed to sit on ice for 30 min. Cell lysates were generated during a 24 h incubation period with cisplatin and the investigated drugs at their IC_50_ dosage. In each reaction, a well containing 0.2 mL of 1 x HEPES buffer was filled with 5 mL of reconstituted AcDEVD-AMC, a synthetic tetrapeptide fluorogenic substrate for caspase-3 activity. Each reaction/well received 20 μL of cell lysate. The reaction mixtures were incubated at 37 °C for 1 h. Using a microplate reader (Perkin-Elmer/Victor/X3) with an excitation wavelength of 380 nm and an emission wavelength of 460 nm, the amount of AMC released from Ac-DEVD-AMC was quantified. When compared with controls, apoptotic cell lysates containing active caspase-3 produced a significant emission. Furthermore, the AMC emission of nonapoptotic control cell lysates was observed to be 100%, and the emissions of other cell lysates were assessed in accordance with the emissions of the control cells. For all doses, duplicate wells were used. This experiment was performed according to Yurttaş et al.^52^

#### 4.2.5 Mitochondrial Membrane Depolarization

Staining of cells with JC-1 was realized according to the manufacturer’s recommendations of BD, Pharmingen Flow cytometry kit. After the most active compounds were determined by the MTT method, the mitochondrial membrane integrity of the compounds on A549 cells was established based on their IC_50_ concentrations. For this purpose, cells were seeded at an optimal density in six-well plates (not exceeding 1 × 10^6^/mL cells). The cells were then incubated with the substances to be tested at the appropriate concentration and time. After the treatment, each cell suspension was taken into a 15 mL polystyrene centrifuge tube and the cells were centrifuged at 400*g* for 5 min and removed from the supernatant. 0.5 mL of a freshly prepared working solution was added to each pellet, and the solution was vortexed. The test cells were soaked in a JC-1 working solution at 37 °C for 10–15 min, and the cells were washed twice. In the first wash, 2 mL of 1X assay buffer was added and the cells were suspended sensitively. Then, the cells were centrifuged at 400*g* for 5 min and the supernatant was removed, carefully. In the second wash, 1 mL of 1X assay buffer was added and vortexed. After the cells were centrifuged at 400*g* for 5 min, each cell pellet was suspended in 0.5 mL 1X assay buffer and vortexed. Finally, the cell was analyzed by a flow cytometer. Cisplatin was used as a standard control, and the results were compared with this positive control.^53^

#### 4.2.6 Cell Cycle Analysis

Following a 24 h incubation period with the compounds, a cell cycle analysis measurement methodology was implemented in compliance with the manufacturer’s instructions for A549 cells (BD, Biosciences). Next, the cells were immersed in citrate buffer for a short while. At room temperature, the cells were centrifuged for 5 min at 400 g. After the supernatant was decanted, 250 μL of solution A was added to the pellet, and it was allowed to sit at room temperature for 10 min. Solution B (200 μL) was then added, carefully mixed, and incubated at room temperature for 10 min. Solution C (200 μL) was then added. It was gently mixed, allowed to stand at 4 °C for 10 min in the dark, and then examined using BD Bioscience’s MODFID software on a flow cytometer. This method was applied in our previous study.^54^

#### 4.2.7 MMP-9 Inhibition

As described in our previous study,^36^ the method was applied in exactly the same manner. EnzoLife Sciences Inc. provided MMP-9 colorimetric kits (Farmingdale, New York, NY, USA). The MMP Colorimetric Drug Discovery Kits are a comprehensive test technique that uses a thiopeptide (Ac-PLG-[2-mercapto-4-methyl-pentanoyl]-LG-OC_2_H_5_) as a chromogenic substrate to screen MMP inhibitors. Using a microplate reader (BioTek, PowerWave, Gen5 software, Winooski, VT, USA) at room temperature, the UV absorbance was measured at 412 nm. NNGH was employed as an inhibitor of control.

### 4.3 ADME Parameters

Predictions of the ADME properties of the final compounds **4a**–**4l** were identified using SwissADME software.^7,55−57^

### 4.4 Docking Studies

The Protein Data Bank Web site provided the crystal structure of the MMP-9 enzyme (PDBID: 5I12). Schrodinger’s Maestro molecular modeling tool was used for the synthesis of proteins and ligands, grid creation, docking, and visualization experiments.^58,59^

The crystal structure was cleared of water molecules. At the protonation process, the ligand was adjusted to the physiological pH (pH = 7.4). In simulations of molecular docking: The Glide/SP docking techniques were utilized to anticipate the compound **4h** topologies at the active site of target structures and then **4h** was docked to the active site of MMP-9 cavity.
